# Urogenital schistosomiasis burden in school-aged children in Tiko, Cameroon: a cross-sectional study on prevalence, intensity, knowledge and risk factors

**DOI:** 10.1186/s41182-021-00362-8

**Published:** 2021-09-16

**Authors:** Irene Ule Ngole Sumbele, Doris Bennen Tabi, Rene Ning Teh, Anne Longdoh Njunda

**Affiliations:** 1 Department of Zoology and Animal Physiology, University of Buea, Buea, Cameroon; 2 Department of Medical Laboratory Science, University of Buea, Buea, Cameroon; 3 Department of Microbiology and Immunology, College of Veterinary Medicine, Cornell University, Ithaca, NY USA

**Keywords:** Urogenital schistosomiasis, Prevalence, Risk factors, Knowledge, School-aged children

## Abstract

**Background:**

This study aimed at determining urogenital schistosomiasis (UGS) prevalence, intensity, knowledge and risk factors in school-aged children (SAC) in the new endemic focus of Tiko, Cameroon.

**Methods:**

A cross-sectional study including 389 SAC of both sexes aged 5–15 years was carried out between April and June 2018. A structured questionnaire was used to collect demographic data, clinical and predisposing factors. Urine sample collected was used to detect *Schistosoma haematobium* eggs by filtration technique and microhaematuria by Heme dipstick COMBI 11. Logistic regression model was used to determine risk factors of UGS.

**Results:**

The overall prevalence of UGS was 37.0% (CI 32.4–41.9) and 32.6% (CI 28.2–37.5) were positive by egg excretion while 24.4% (CI 20.4–28.9) by haematuria. *S. haematobium* egg excretion and haematuria were significantly higher in males (*P* = 0.016; *P* = 0.049) and children 12–15 years old (*P* = 0.009; *P* = 0.002), respectively. The mean number of eggs per 10 mL of urine was 77.6 (10.2) and ranged from 2 to 400. The proportion of light intensity of infection was higher (67.7%, CI 59.2–75.2) with no significant differences by sex, age and residence. However, the older children were more heavily infected when compared to the younger children, who had more of light infection. Overall, the mean knowledge score 1.42 (CI 1.32–1.51) on a scale of 6, was poor and the proportion of good knowledge of the disease (23.14%, CI 19.2–27.6) was low. Stream water contact (AOR = 4.94; *P* = 0.001) was the only significant risk factor identified.

**Conclusion:**

Urogenital schistosomiasis is of public health concern among SAC in Tiko, Cameroon. Most participants have poor knowledge about the disease, hence education on vector-borne diseases and the avoidance of stream water contact should be implemented.

## Background

Neglected tropical diseases are often associated with serious disabilities and very high mortality rates, with more than a billion people affected and projections of millions of disability-adjusted life years [1]. Schistosomiasis which is a neglected tropical disease, is second to malaria alone amid the vector-borne diseases in terms of public health [2]. It is second to none in terms of prevalence amongst water-borne diseases [3]. A great majority (85%) of cases occur in Africa [4]. Despite the gains in the health care delivery of the past decades, schistosomiasis has prevailed as a health challenge in the tropics and sub-tropics.

Schistosomiasis is a disease of the poor who live in conditions that favour transmission; an insidious disease, poorly recognized at early ages and disabling to men and women during their most productive years [5]. Schistosomiasis is caused by several species of the genus *Schistosoma*. It can be grouped into two categories based on the organ affected—urogenital schistosomiasis and intestinal schistosomiasis. Urogenital schistosomiasis is caused by *Schistosoma haematobium* and intestinal schistosomiasis by any of the following organisms namely, *S. guineensis, S. intercalatum, S. mansoni, S. japonicum, and S. mekongi.*

Urogenital schistosomiasis (UGS) caused by *S. haematobium* is acquired from infested freshwater snails of the *Bulinus* species. Its transmission takes place only where snail vectors are present and where there is contact between the population and infected freshwater sources containing parasites egg which hatch in the water [6]. People become infected when larval forms of the parasite (cercaria) released by freshwater snails penetrate the skin during contact with infested water. In the body, the larvae develop into adult schistosomes and the parasite is found in the venous plexus draining the urinary bladder of humans [7]. Various socio-epidemiological factors are responsible for transmission of the disease amongst which are migration, distance from transmission site and emergence of new foci, which is the main factor in this present case [8].

The characteristic clinical presentation of infection with *S. haematobium* is terminal haematuria, usually associated with increased frequency of micturition and dysuria. The infection is also associated with anaemia [9], nutritional deficiencies and growth retardation [10], adverse effects on cognitive development, decreasing physical activity and school performance as well as work capacity and productivity [11]. Diagnosis is made by finding the characteristic ova in the urine using the filtration techniques.

Alongside malaria, schistosomiasis is also a documented public health problem in Cameroon and contributes to a third of the morbidity among school-age children (SAC) [12, 13]. The mainstay of schistosomiasis control in endemic foci in Cameroon is preventive chemotherapy—the periodic administration of praziquantel to at-risk groups (e.g., school-age children (4–14 years) in schools or through community directed programmes [14]. While this strategy does not prevent infection or reinfection, it reduces morbidity and might also influence transmission [15]. Despite all the efforts put in place by the Ministry of Public Health in Cameroon to curb UGS and to reduce its outbreak, it remains an undiminishing threat with new foci such as Tiko, on the rise, which has not benefitted from this control. Tiko is a semi-urban area with many streams that have been used by the inhabitants for laundry, swimming and fishing, exposing the population to water and vector-borne disease.

Recent report highlights the emergence of urogenital schistosomiasis among SAC and reproductive aged individuals in Likomba, a community in Tiko Health District [16, 17]. It is therefore vital to monitor the variation of intensity of infection and associated morbidity in Likomba and another closer neighbourhood like Upper Costen in the same Health District suspected for UGS, due to the migration of individuals from the conflict-hit areas of Muyuka and Ikata-Likoko, endemic with UGS [18, 19]. The consequences of the ongoing civil strife in the North West and South West Regions of Cameroon have been enormous [19, 20], with over half a million people displaced the majority of whom are women and children [21]*.* The main objective of this study was to generate baseline data on the prevalence of UGS in SAC in Tiko; raise awareness through evaluation of knowledge of the disease as well as identify risk factors to enable strategic planning of control programmes in this endemic focus.

## Materials and methods

### Study area and participants

**Fig. 1:**
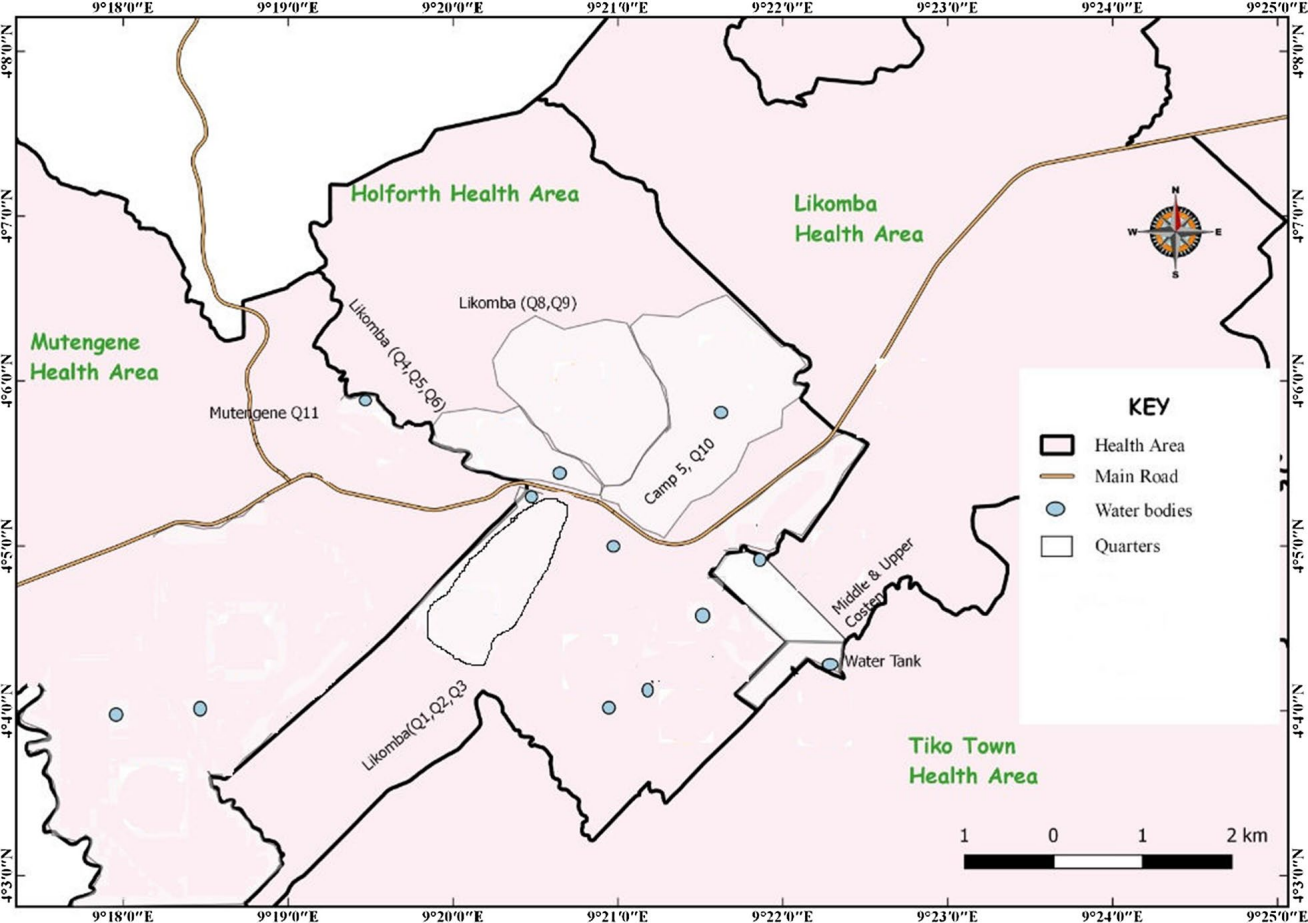
Map showing the study sites (Likomba and Upper Costen) and water bodies

The study was carried out in Likomba and Upper Costen in the Tiko Health District in the South West Region of Cameroon. The map showing the study sites and existing water bodies is represented in Fig. 1. A detailed description of the study area is given in Anguh et al*.* [16]. Tiko municipality has a coastal equatorial climate. There is poor urban planning with some houses having neither toilets nor water, while some share toilets in a group. Defecation is done in empty plots of land common in the area. Due to influx of internally displaced persons from conflict-hit areas and conditions of poor personal hygiene and environmental sanitation, diseases like schistosomiasis are bound to be on the rise. The town partly hosts the CDC (Cameroon Development Co-operation) industrial farms, which produces rubber, banana and palm oil.

The study population included SAC of both sexes aged 5–15 years whose parent or guardian consented through signing an assent form to their participation in the study. Participants were recruited if they met the eligibility criteria of being ≥ 5 years and < 16 years, lived in the area for at least a year before the commencement of the study, demonstrated the willingness to participate in the study and could understand English/French or could respond to questions asked in pidgin.

### Study design and sampling

This community-based cross-sectional study was carried out in Tiko in the South West Region of Cameroon within the period of April to May 2018. SAC from Upper Costen and Likomba were recruited by a convenience sampling method as they presented themselves to the research team following education through community relay agents. Children whose parent/guardian signed the informed consent/assent form following education were enrolled. Data on socio-demographics, clinical, perceptions and knowledge on UGS, were collected using a simple structured questionnaire. Confidentiality was taken into consideration as participants responded to the questions in the questionnaire. Subsequently, each participant was given a sterile, wide mouthed, screw-capped plastic container carrying their identification numbers and instructions on how to collect the urine samples.

### Sample size determination

The sample size of the study population was determined using the formula *n* = *Z*^2^*pq*/*d*^2^ [22], where n was the sample size required; *Z* was 1.96, which is the standard normal deviate (for a 95% confidence interval, CI); *p* was 38%, the proportion of UGS prevalence reported in the area [14]; *q* was 1−*p*, the proportion of UGS negative; and d was 0.05, the acceptable error willing to be committed. The optimum sample size was estimated to be 362. A total of 389 participants were enrolled into the study in anticipation of incomplete data entry, loss of data, voluntary withdrawal and for superior precision.

### Data collection

A simple semi-structured questionnaire was pretested in urogenital schistosomiasis endemic focus of Muyuka. The respondents selected for pre-testing of the questionnaire consisted of 50 parents/caregivers above 18 years of age, of both sexes and different levels of education. The questionnaire was modified accordingly before being administered. The information collected included demographic, socio-economic, environmental background, personal hygiene, clinical signs and symptoms of UGS; household cleanliness including availability of functioning toilets, use of pipe-borne water, wearing shoes when outside the house and washing of hands. The children's parents or adult guardians were the respondents and those who could not read or write were aided via a face-to-face interview.

### Urine sample collection and processing

About 20 ml of midstream urine sample was collected into a clean, dry, open mouth, capped and labelled container after a brisk exercise between 10am and 2 pm. The urine sample was immediately checked for microhaematuria using the Heme dipstick COMBI 11 by dipping it in the urine and reading after 120 s for colour changes following the manufacturers instruction. The samples were preserved in a cooler (temperature: 2–8 ℃) and transported to the Malaria Research Laboratory of the University of Buea for processing and examination. All samples were examined no later than 12 h after collection. Filtration technique was used as a quantitative test following standard procedure [23]. Briefly, using blunt-ended forceps, a polycarbonate filter (STERLITECH Corporation USA) was carefully placed in the filter support of the filter holder. The filter holder was reassembled and attached to the end of a 10-mL syringe. The syringe was filled to the 10-mL mark with well mixed urine and the urine sample passed through the filter. With the aid of blunt forceps, the filter was removed after unscrewing the filter holder and transferred face upwards, that is eggs on the surface on a microscope slide. A drop of iodine was added to the slide and covered with cover glass. Using the ×10 objective the entire filter was examined systematically for *S. haematobium* egg and the total number of eggs seen was counted and recorded per 10 mL of urine. The intensity of infection was reported as light infection (1–49 eggs/10 mL of urine) while 50 eggs and above/10 mL of urine was classified as heavy infection [24].

### Data analysis

Data were double entered by two different researchers into a spreadsheet of SPSS version 25.0. Then a third researcher crosschecked the two data sets for accuracy and created a single data set for data analysis. Socio-demographic characteristics were treated as categorical variables and presented as frequencies and percentages. The prevalence of UGS was presented as proportions. Intensity of UGS was expressed as proportion of light and heavy infection and Chi-square (*χ*^2^) was used to examine the association between infection intensity and demographic factors (sex and age). A multivariate logistic regression analysis was used to identify the risk factors that were significantly associated with UGS. Odds ratio (OR), adjusted odds ratio (AOR) and a 95% confidence interval (CI) were computed for each variable. The level of statistical significance was set as *P* < 0.05.

Mean knowledge scores and proportions were used to determine the level of good knowledge of participants on UGS. For knowledge scoring and analysis, a total of six item or questions were included in the knowledge section which included elementary knowledge on the cause, mode of transmission and prevention of schistosomiasis. For these six knowledge questions, the maximum attainable score was ‘6’ and the minimum score was ‘0’. The level of knowledge was classified according to each respondent’s score. Good knowledge corresponds to a score greater than 3 (i.e. participants with more than 3 correct responses out of the 6 knowledge questions) while poor knowledge corresponded to a score less than 3 (i.e. participants with less than 3 correct responses out of the 6 knowledge questions).

### Definition of end points

UGS: presence of schistosomiasis when diagnosed positive by microscopic examination and/or urine reagent strips.

Haematuria: presence of schistosomiasis when diagnosed positive by urine reagent strips.

## Results

### Socio-demographic characteristics of participants

A total of 389 SAC (53.4% males and 46.6% females) aged between 5 and 15 years, with a mean age of 9.4 ± 3.1 years were enrolled in this study. Of these, 43.4% resided in Upper Costen and 56.6% resided in Likomba. Majority (75.6%) of the participants were in primary school and only one-third (32.7%) of the households owned a latrine (Table 1).

**Table 1:** Socio-demographic characteristics of participants

Variable	Category	Frequency	Percent (%)
Gender	Male	208	53.47
	Female	181	46.53
	5–8	156	40.10
Age (years)	9–11	121	31.10
	12–15	112	28.79
Mean age (years) ± SD	9.4 ± 3.4		
Educational level	Primary education	294	75.58
	Secondary education	95	24.42
Household owns a latrine	Yes	127	32.65
	No	262	67.35
Area of residence	Upper Costen	169	43.4
	Likomba	220	56.6

### Prevalence and intensity

Out of the 389 SAC examined, 32.6% (CI 28.2–37.5) were *S. haematobium* egg positive with similar prevalence in Upper Costen (31.4%, CI 24.9–38.7) and Likomba (33.6%, CI 27.7–40.1). However, the prevalence was significantly higher in males (38.0%, CI 31.7–44.7) and those 12–15 years (42.0%, CI 33.2–51.2) old than their counterparts as shown in Table 2.

**Table 2:** Prevalence and intensity of *S. haematobium* egg and haematuria by sex, age and residence

Parameter and category	*N*	*S. haematobium* egg prevalence % (*n*)	*S. haematobium* egg intensity		Haematuria prevalence
			Light	Heavy	
Overall	389	32.6% (127)	67.7 (86)	32.3 (41)	24.4 (95)
Sex					
Male	208	38.0 (79)	64.6 (51)	35.4 (28)	28.4 (59)
Female	181	26.5 (48)	72.9 (35)	27.1 (13)	19.9 (36)
χ ^2^		5.782	0.954		3.87
*P* value		**0.016**	0.329		**0.049**
Age (years)					
5–8	156	24.4 (38)	68.4 (26)	31.6 (12)	17.3 (27)
9–11	121	34.7 (42)	69.0 (29)	31.0 (13)	23.1 (28)
12–15	112	42.0 (47)	66.0 (31)	34.0 (16)	35.7 (40)
χ ^2^		9.529	0.109		12.123
*P* value		**0.009**	0.947		**0.002**
Area of residence					
Upper Costen	169	31.4 (53)	67.9 (36)	32.1 (17)	22.5 (38)
Likomba	220	33.6 (74)	56.8 (42)	43.2 (32)	25.9 (57)
χ ^2^		0.225	1.625		0.607
*P* value		0.358	0.202		0.436

Light intensity of infection, 1–49 eggs/10 mL of urine; heavy intensity of infection,  ≥ 50 eggs/10 mL of urine. *P* values in bold are statistically significant

**Fig. 2:**
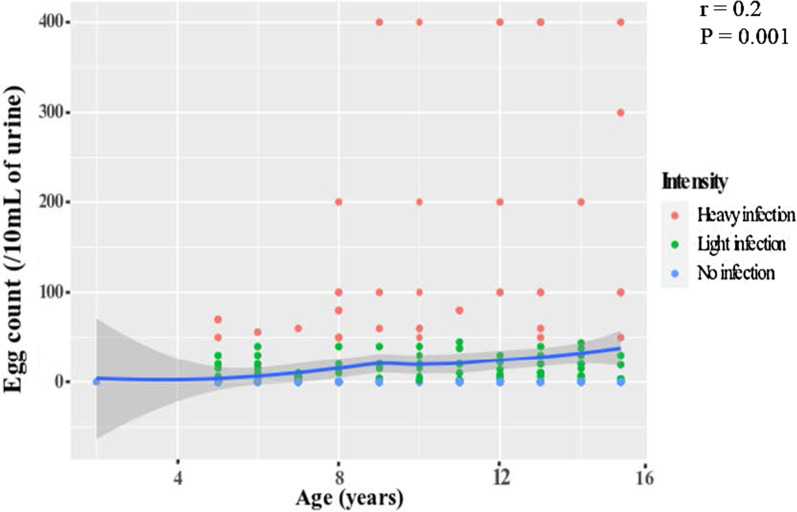
Correlations between egg count and age of the participants

The mean (SD) number of eggs per 10 mL of urine was 77.6 (10.2) and ranged from 2 to 400. The proportion of SAC with light intensity of infection (1–49 eggs/10 mL of urine) was higher (67.7%, CI 59.2–75.2) than that with heavy intensity infection (≥ 50 eggs/10 mL of urine). No significant variations in eggs excretion intensity were observed between sexes (*P* = 0.329), age groups (*P* = 0.947) and residence (*P* = 0.202) (Table 2). However, the older children were more heavily infected when compared to the younger children, who had more of light infection (*r* = 0.2, *P* = 0.001) as shown in Fig. 2.

Haematuria was common in 24.4% (CI 20.4–28.9) of the study population with statistically significant variation by sex (*P* = 0.049) and age (*P* = 0.002). The prevalence was significantly higher in males than females (28.5%, CI 22.7–34.8 vs 19.9%, CI 14.7–26.3) and increased with age from 17.3% (CI 12.2–24.0) in those 5–8 years old to 35.7% (CI 27.5–44.9) in those 12–15 years old as revealed in Table 2.

### Participant knowledge of urogenital schistosomiasis (UGS)

The proportion of participants with good knowledge on UGS was 23.1% (CI 19.2–27.6). Only 19.3% (CI 15.7–23.5) of the participants had ever heard about UGS with the school being the main source of information 12.1% (CI 9.2–15.7). Barely 19.3% (CI 15.7–23.5) of the participants knew UGS was transmitted via water. Concerning the signs and symptoms, 11.1% (CI 8.3–14.6) of them identified haematuria as the main symptom of UGS and 54.3% (CI 49.3–59.1) were aware the disease could be treated. Regarding methods of prevention, majority (82.8%, CI 84.3–90.8) had no knowledge on how the disease could be prevented while 7.5% (CI 5.2–10.5) said it could be prevented by not swimming in water bodies as shown in Table 3.

**Table 3:** Proportion of participants’ awareness of urogenital schistosomiasis

Question	Response	Frequency	Percent (%)
Have you ever heard about schistosomiasis?	Yes	75	19.3
	No	314	80.7
Source of information about schistosomiasis	School	47	12.1
	Out of school	27	6.9
	Do not know	315	81.0
How is the disease transmitted	Water	75	19.3
	Food	0	0
	Do not know	314	80.7
What are the signs of schistosomiasis?	Haematuria	43	11.1
	Painful urination	4	1.0
	Do not know	342	87.9
Do you know that the disease can be treated?	Yes	211	54.3
	No	178	45.7
What do you think should be done to prevent this disease?	Treatment	38	9.7
	Stop swimming	29	7.5
	Do not know	322	82.8
Knowledge proportion	Good	90	23.1
	Poor	299	76.8

**Fig. 3:**
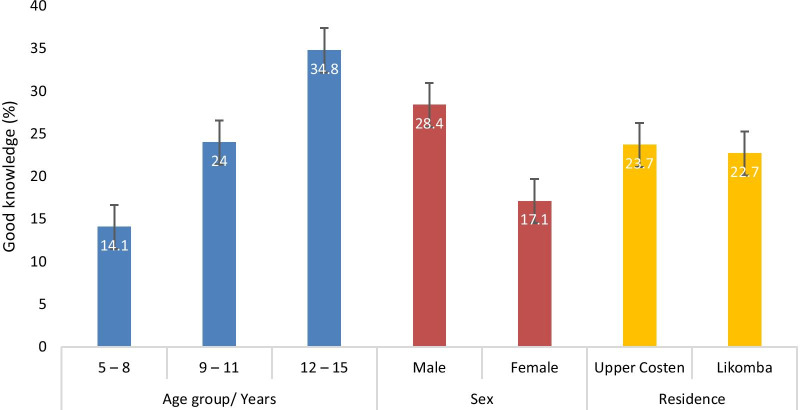
Proportion of participants with good knowledge by age, sex and area of residence

The combined knowledge score was classified according to each respondent’s score. The mean knowledge score of the participants was 1.42 (CI 1.32–1.51) on a scale of 6. While the proportion of participants with good knowledge on UGS was comparable between those from Upper Costen and Likomba, the proportion significantly increased with age (χ2 = 15.805 *P* < 0.001) with SAC 12–15 years old (34.8%, CI 26.6–44.0) being more knowledgeable than their counterparts as shown in Fig. 3. In addition, significantly higher (χ2 = 6.874 *P* = 0.009) proportion of males (28.4%, CI 22.7–34.8) were more knowledgeable of the disease than females.

### Risk factors associated with urogenital schistosomiasis in SAC

Overall, the prevalence of UGS was 37.0% (144/389) (CI 32.4–41.9). The bivariate and the multivariate logistic regression model with UGS as the dependent variable and age, gender, educational level of parents, knowledge on UGS, source of water for domestic use and water contact, as independent variables is presented in Table 4. In the bivariate analysis, SAC aged 12–15 years (crude odds ratio (COR) = 2.37, *P* = 0.001), being male (COR = 1.58, *P* = 0.033), having good knowledge of UGS (COR = 2.42, *P* = 0.001), using water from stream for domestic purpose (COR = 1.60, *P* = 0.033) and stream water contact (COR = 6.19, *P* = 0.001) were identified as significant risk factors of UGS. On the other hand, in the multivariate analysis, while most variables were potentially risk factors based on the adjusted odds ratio (AOR), only that of water contact was statistically significant (AOR = 4.94, *P* = 0.001) as shown in Table 4.

**Table 4:** Bivariate and multivariate models showing factors associated with urogenital schistosomiasis prevalence

Variable	*N*	Urogenital schistosomiasis prevalence (*n*)	Bivariate analysis		Multivariate analysis	
			COR (95% CI)	*P*-value	AOR (95% CI)	*P*-value
Age group (years)						
5–8	156	28.21 (44)	Reference		Reference	
9–11	121	38.02 (46)	1.56 (0.94–2.59)	0.084	1.18 (0.68–2.04)	0.558
12–15	112	48.21 (54)	2.37 (1.42–3.94)	**0.001**	1.67 (0.77–3.64)	0.197
Gender						
Female	181	31.49 (57)	Reference		Reference	
Male	207	42.03 (87)	1.58 (1.04–2.40)	**0.033**	1.17 (0.74–1.85)	0.499
Educational level of parents						
Secondary	294	34.69 (102)	Reference		Reference	
Primary	95	44.21 (42)	1.49 (0.93–2.39)	0.096	1.23 (0.59–2.58)	0.587
Knowledge on schistosomiasis						
Poor	299	32.11 (96)	Reference		Reference	
Good	90	53.33 (48)	2.42 (1.50–3.91)	**0.001**	1.84 (1.10–3.08)	0.059
Source of water for domestic use						
Tap	196	32.14 (63)	Reference		Reference	
Stream	160	43.13 (69)	1.60 (1.04–2.47)	**0.033**	1.36 (0.85–2.17)	0.195
Well	33	36.36 (12)	1.20 (1.21–2.61)	0.633	1.12 (0.49–2.59)	0.782
Stream water contact						
No	112	12.50 (14)	Reference		Reference	
Yes	277	46.93 (130)	6.19 (3.37–11.36)	**0.001**	4.94 (2.63–9.29)	**0.001**

UGS, egg/haematuria positive; COR, crude odds ratio; AOR, adjusted odds ratio; *P* values in bold are statistically significant

## Discussion

Due to lack of effective intervention programmes on the control and prevention of urogenital schistosomiasis, there is continuous resurgence and outbreaks. This study was carried out in the new focus in Tiko in the Mount Cameroon area with the main objective to generate baseline data on the prevalence of urogenital schistosomiasis in SAC, raise awareness through evaluation of knowledge of the disease as well as identify risk factors to enable strategic planning of control programmes to make use of the limited resources.

The prevalence of urogenital schistosomiasis in this study is higher than that reported in Maroua, in the Far North of Cameroon [25], and other communities in the Mount Cameroon area such as in Muyuka [26], Barombi Mbo [27], Ikata-Likoko area [28] and Kumba [29]. On the other hand, the prevalence is lower than that reported in other foci in regions like Munyenge [30], Kotto Barombi [31, 32] and Magba [33]. Although this study area was regularly targeted for control of geohelminths, schistosomiasis control was never given due attention since the focus is relatively new as a result of migration of infected population from the conflict-hit endemic foci in the Mount Cameroon area to the locality, probably accounting for the observed prevalence [16]. In addition, the other study areas are essentially rural areas with only community health care with limited pipe-borne water supply for domestic needs while Tiko is a semi-urban setting with improved social amenities. Even though urbanization reduces transmission points and the creation of modern water points limits the frequency of human water contacts as noted by Njiokou et al. [34], this has not had much of an impact in the communities studied due to their proximity to stream water sources that could be used for domestic purposes at no cost, rather than using pipe-borne water which is paid for.

Findings from the study demonstrated that urogenital schistosomiasis was significantly more common in males. This could be attributed to the higher tendencies of water contact through swimming, playing, and engagement in other domestic activities like fetching water and laundry in infested water bodies. This higher prevalence of schistosomiasis among males is in line with other studies performed elsewhere [35–37]. These findings are however contradictory to those of Sumbele et al*.* [26], Ntonifor et al. [38] and Ndamukong et al. [29], in other communities of the Mount Cameroon area in which the infection was more common in females.

Infection with *S. haematobium* was observed to be more common in children above 11 years of age than those below 10 years. The finding of higher prevalence in children above 11 years is in line with the study by Ntonifor et al. [30] and Kimbi et al. [39]. This however contradicts the findings of Njunda et al. [33] and Ebai et al. [28] who reported a higher prevalence of urogenital schistosomiasis in children 10 years and below, which could be attributed to the different behavioural pattern and cultural practices of the different study populations. As children grow older in semi-urban communities, the boys take up more difficult domestic tasks of fetching water and laundry and find pleasure in swimming in the stream for longer periods than the females.

The prevalence of urogenital schistosomiasis was not significantly different in school-aged children residing at Likomba and Upper Costen. The lack of such a difference might be due to the presence of several accessible water bodies at the centre of the town near residential areas where many children are always found bathing, swimming and doing laundry. This observation is in line with Kimbi et al*.* [39] and Campbell et al. [27], who also reported higher prevalence of urogenital schistosomiasis in children living near transmission sites.

The mean knowledge score and proportion of good knowledge of the disease is low with more males than females being knowledgeable of the disease in line with studies conducted by Kamga et al*.* [40] and Abdulkareem et al*.* [41]. This observation contradicts that of Aron et al. [42] in Tanzania who reported a higher proportion (92.4%) of participants with knowledge on urogenital schistosomiasis attributed to its high occurrence. Alternatively, the poor knowledge of the disease in the Tiko endemic focus may be linked to the relatively short period of existence of the disease in the area hence, many children may not have experienced haematuria at any point in time in their life that would have probably raised awareness in the population unlike in highly endemic areas. Furthermore, consistent with other observations, older children were more knowledgeable of the disease than those younger [43]. These older children who are mostly on the course of secondary education can carry out more independent reading and quest for more information if need arises than their counterparts thus asserting that education improves knowledge. Interestingly, older children although more knowledgeable of the disease had the highest prevalence. This could probably be that the older children were not putting into practice their knowledge of schistosomiasis especially in the different preventive strategies of successful schistosomiasis prevention and control. Generally, older children take up more difficult domestic tasks of fetching water and laundry and find pleasure in swimming. Nevertheless, it is important to build and maintain broader understanding of schistosomiasis and its preventive strategies, especially environmental management, role of the vector and ways to control it. Therefore, well planned and locally sensitive ongoing public educational interventions are essential to educate and help communities to improve and sustain schistosomiasis knowledge and desired protective behaviour while addressing external factors such as environmental management.

Although male participants, children older than 11 years, having good knowledge of the disease, stream as a source of water for domestic use and stream water contact were 1.6-, 2.4-, 2.4-, 1.6- and 6.1-fold, respectively, more at risk of having urogenital schistosomiasis in the bivariate model, only contact with infested water body was identified as a risk factor in the multivariate model. This observation is not atypical as intense water contact activities have been attributed to the prevalence of infection elsewhere [41]. Infection occurs when cercaria, the larval form of the parasite is released by freshwater snails of the Genus *Bulinus,* penetrates the skin during contact with infested water. Hence, avoiding domestic and recreational activities such as swimming or fishing in infested water [44] may curb the spread of the disease.

While the design of the study gives a snapshot of information on the burden of urogenital schistosomiasis in school-aged children in this new endemic focus of Tiko, these findings are invaluable to health authorities in programming and re-structuring to include this area in the existing control programme in the country. The lack of detailed malacological information on the various streams existing in the community and detail assessment of intensity of water contact activities carried out is a major limitation to the study that demands further investigation to give a holistic picture on the epidemiology of urogenital schistosomiasis in the area.

## Conclusions

These findings suggest that urogenital schistosomiasis is of public health concern among SAC in Tiko. The burden of infection is higher in males and children 12–15 years with most of the participants having a poor knowledge about the disease. Hence, the population should be educated on vector-borne diseases and the avoidance of stream water contact which is the main risk factor identified in the study. Despite considerable efforts to scale-up activities to encompass all the regions in the country, coverage remains inadequate especially with the spread to other new foci, thus preventing the desired goals to be achieved. Hence, the preventive chemotherapy with praziquantel employed in other endemic focus in the region, should be supplemented with the provision of potable water in these communities to limit water contact activities in infested streams.

## Data Availability

All datasets on which the conclusions of the research rely are presented in this paper. However, data are available from the corresponding author on reasonable request.

## Abbreviations

UGS: Urogenital schistosomiasis
SAC: School-aged children
χ^2^: Chi-square
COR: Crude odds ratio
AOR: Adjusted odds ratio
CI: Confidence interval
